# High efficacy of intensive immunochemotherapy for primary mediastinal B-cell lymphoma with prolonged follow up

**DOI:** 10.1038/s41598-022-14067-3

**Published:** 2022-06-22

**Authors:** Joanna Romejko-Jarosinska, Beata Ostrowska, Anna Dabrowska-Iwanicka, Katarzyna Domanska-Czyz, Grzegorz Rymkiewicz, Ewa Paszkiewicz-Kozik, Robert Konecki, Anna Borawska, Agnieszka Druzd-Sitek, Elzbieta Lampka, Wlodzimierz Osiadacz, Michal Osowiecki, Lidia Popławska, Monika Swierkowska, Lukasz Targonski, Joanna Tajer, Grazyna Lapinska, Malwina Smorczewska, Jan Walewski

**Affiliations:** 1 Department of Lymphoid Malignancies, Maria Sklodowska-Curie National Research Institute of Oncology, 5 WK Roentgen Str, 02-781 Warsaw, Poland; 2 Department of Pathology and Laboratory Diagnostics, Maria Sklodowska-Curie National Research Institute of Oncology, Warsaw, Poland; 3 Department of Endocrine Oncology and Nuclear Medicine, Maria Sklodowska-Curie National Research Institute of Oncology, Warsaw, Poland; 4 Department of Radiology, Maria Sklodowska-Curie National Research Institute of Oncology, 02-781 Warsaw, Poland

**Keywords:** Diseases, Oncology

## Abstract

Primary mediastinal B-cell lymphoma (PMBL) is currently curable in 85–95% of patients. Treatment regimens frequently used include RCHOP ± radiotherapy, DAEPOCH-R, or occasionally more intensive protocols. Here we present results of treatment of 124 patients with PMBL over a period between 2004 and 2017 with the use of a protocol designed for aggressive B-cell lymphoma GMALL/B-ALL/NHL2002 including 6 cycles of alternating immunochemotherapy with intermediate-dose methotrexate in each cycle, and reduced total doxorubicin dose (100 mg/m^2^ for whole treatment). Majority of patients (77%) received consolidative radiotherapy. A median (range) age of patients was 30 (18–59) years, and 60% were female. With a median (range) follow up of 9 (1–17) years, 5-year overall survival (OS) and 5-year progression free survival (PFS) were 94% and 92%, respectively. Positron emission tomography—computed tomography (PET-CT) results at the end of chemotherapy were predictive for outcome: OS and PFS at 5 year were 96% and 94% in PET-CT negative patients, respectively, and 70% and 70% in PET-CT-positive patients (p = 0.004 for OS, p = 0.01 for PFS). Eight (6%) patients had recurrent/refractory disease, however, no central nervous system (CNS) relapse was observed. Acute toxicity included pancytopenia grade 3/4, neutropenic fever, and treatment related mortality rate of 0.8%. Second malignancies and late cardiotoxicity occurred in 2.4% and 2.4% of patients, respectively. Intensive alternating immunochemotherapy protocol GMALL/B-ALL/NHL2002 is curative for more than 90% of PMBL patients and late toxicity in young patients is moderated. The attenuated dose of doxorubicin and intermediate dose of methotrexate may contribute to low incidence of late cardiotoxicity and effective CNS prophylaxis.

## Introduction

Primary mediastinal large (thymic) B cell lymphoma (PMBL) is an aggressive disease with unique pathological, molecular and clinical features^1^. It accounts 2–4% of lymphoma^1–3^. Recent studies based on targeted sequencing and mutational profile suggest common molecular features of PMBL and non-mediastinal DLBCL tumours as well as relation to classical Hodgkin lymphoma^2,3^. Due to rarity of the disease and absence of randomized studies there is general uncertainty on the optimal treatment approach. Our knowledge is based primary on retrospective reports with a limited number of patients. Generally, R-CHOP with or without RT is the most widely used regimen for PMBL^4–10^ with a 5-year PFS and OS of 77–95% and 84–98%, respectively. Recent studies suggested that RT can be safely abandoned if end-of treatment positron emission tomography (EOT-PET) is negative^7,11^. A continuous infusion variant of CHOP chemotherapy enriched in etoposide, i.e. DA-EPOCH-R regimen recently promoted by the group from National Cancer Institute, USA, was implemented in a number of North American centers based on results of phase II study of 51 PMBL patients with 5-year event free survival and 5-year OS of 93% and 97%, respectively^11^. Notably, patients were not exposed to radiotherapy^11^. However, a recent study of DA-EPOCH-R in 46 children and adolescent (median age of 15.4 years) with PMBL resulted in 4-year EFS of 69.6% that was not different from the rate observed historically^12^.

A protocol developed for Burkitt lymphoma by the German Multicenter Adult ALL Group (GMALL) involving intermediate to high dose antimetabolites and fractionated alkylating agents followed by mediastinal radiotherapy (GMALL/B-ALL/NHL 2002)^13–15^ included PMBL patients based on general experience of poor patient outcome in case of relapse or disease progression. Additionally, in GMALL protocol the doxorubicine-dose was limited to reduce cardiac complications in cured young patients. Based on encouraging preliminary results with more than 90% of PMBL patients achieving long-term survival, no treatment related mortality (TRM), and no relapses in central nervous system (CNS)^15^, GMALL protocol was implemented for PMBL patients at our institution. Here we present results of treatment of 124 consecutive newly diagnosed PMBL patients treated with intensive GMALL protocol between 2004 and 2017.

## Patients and methods

The retrospective analysis included all newly diagnosed PMBL patients treated at the National Research Institute of Oncology in Warsaw between April 2004 and December 2017. The diagnosis of PMBL was established according to the 2016 WHO classification criteria^1,16^ by the same reference hematopathologist, routinely undertaking histopathological and immunohistochemical examinations including mediastinal tumors and performing flow cytometry in some cases as previously described^16^. All consecutive patients received the GMALL/B-ALL/NHL2002 therapy developed for patients aged 18–55^17^. The treatment protocol was approved by the Institutional Ethical Review Board of National Research Institute of Oncology in Warsaw (23/2004) on 1st of April 2004 in accordance with the Declaration of Helsinki. Informed consent for GMALL/B-ALL/NHL2002 therapy was obtained from all patients. Patients were previously untreated (except short-term administration steroids less than 7 days, single administration of cyclophosphamide/vincristine, one cycle of CHOP ± R), and received no radiotherapy. Patients were not included after the end of 2017 to allow for at least 3-year follow-up.

The GMALL/B-ALL/NHL/2002 protocol^17^ for patients aged 18–55 consisted of the prephase containing cyclophosphamide 200 mg/m^2^ i.v. days 1–5 and prednisone 60 mg/m^2^ p.o. days 1–5 or 1 cycle of CHOP ± R. Full therapy included 6 alternating blocks A1, B1, C,1 A2, B2, C2. Each block contained rituximab 375 mg/m^2^ i.v. day 1, methotrexate 1500 mg/m^2^ day 1, dexamethasone 10 mg/m^2^ p.o./i.v. days 1–5. Block A included ifosfamide 800 mg/m^2^ i.v. days 1–5, vincristine 1.4 mg/m^2^ i.v. day 1 (max 2 mg), teniposide 100 mg/m^2^ i.v. days 4–5, cytarabine 300 mg/m^2^ days 4–5. Block B included cyclophosphamide 200 mg/m^2^ i.v. days 1–5, vincristine 1.4 mg/m^2^ i.v. (max 2 mg), doxorubicin 25 mg/m^2^ i.v. days 4–5. Block C included etoposide 250 mg/m^2^ i.v. days 4–5, cytarabine 2000 mg/m^2^ i.v. every 12 h day 5. CNS triple prophylaxis (methotrexate, cytarbine dexamethasone) was given in blocks A and B. The protocol involved consolidative radiotherapy 30–39 Gy to mediastinum for all patients, however, decision was left to treating physician.

Standard staging included blood examination, whole body computed tomography (WB-CT), bone marrow biopsy, echocardiography for cardiac function assessment and cytologic analysis of cerebrospinal fluid. WB-CT scans were performed during and after treatment to assess interim and the end of chemotherapy response. PET-CT imaging was routinely performed after 2010. PET-CT response assessment was based on 5 point Deauville score. Score 1–3 was considered “negative” or metabolic complete remission (mCR), score 4–5 was considered “positive” or active disease.

PFS was calculated from the date of the first treatment to the date of progression, relapse, death of any cause, or the last date of follow up in patients without relapse. OS was calculated from the first date of treatment to the date of death or the last date of follow up. Overall response to therapy was evaluated according to the International Working Group Recommendations for Response Criteria for NHL^18,19^. Toxicity was graded according to the National Cancer Institute Common Criteria for Adverse Events version 4.02. OS and PFS were estimated and plotted using the Kaplan–Meier survival analysis. The associations between dichotomized prognostic factors and PFS/OS were analyzed using log rank (Mantel-Cox) test in univariate analysis. A *P* value < 0.05 was considered statistically significant. The STATISTICA StatSoft.PL v 13.1. package was used to perform analyses.

### Ethical approval

Local ethics committee approved GMALL protocol on 1st of April 2004 (23/2004) in accordance with the Declaration of Helsinki.

## Results

**Figure 1:**
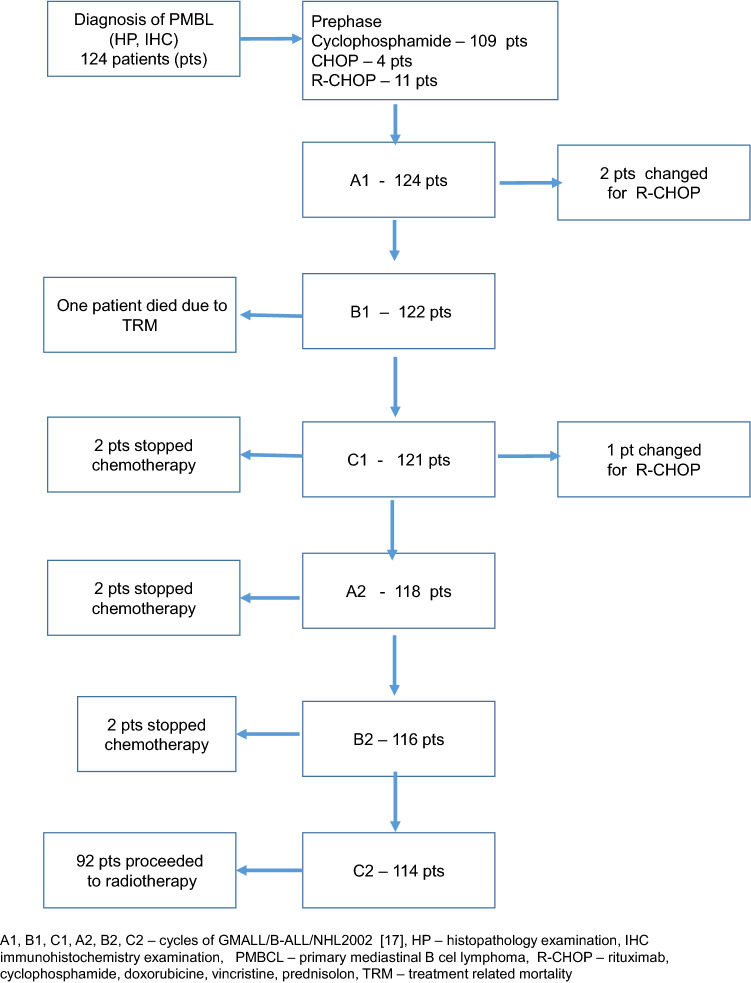
GMALL/B-ALLL/NHL2002 – treatment disposition. A1, B1, C1, A2, B2, C2 – cycles of GMALL/B-ALL/NHL2002^17^.

Between April 2004 and December 2017, 124 consecutive patients were treated with GMALL/B-ALL/NHL2002 protocol. The median age (range) was 30 (18–59). Most of the patients had CS I–II (62%) and bulky mediastinal mass (83%); 21 patients (17%) had ECOG PS ≥ 3. Five patients were initially admitted to the Intensive Care Unit (ICU) due to respiratory insufficiency, and GMALL protocol was initiated in parallel to the intensive care. Patient characteristics at baseline are presented in Table 1. One hundred and nine (89%) patients started with cyclophosphamide prephase. Four patients received one cycle of CHOP and 11 patients was given one cycle of R-CHOP. Out of 124 patients, 114 (92%) received 6 cycles of chemotherapy, 3 patients changed GMALL/B-ALL/HL2002 treatment to R-CHOP, 6 patients discontinued chemotherapy after 3–5 cycles due to physician decision and 1 patient treated at the ICU died after second cycle due to toxicity (Fig. 1). Consolidative radiotherapy at a median (range) dose 36 (30–39) Gy/t was applied to 92 patients (77%): in 70 patients with complete remission (CR) and in 21 with partial remission (PR) after chemotherapy; 78% of irradiated patients had CSI–II.

**Table 1:** Patient characteristics at the diagnosis.

Variable	N = 124 (%)
Median age (range)	30 (18–59)
Female	75 (60)
ECOG performance status ≥ 2	82 (66)
ECOG performance status ≥ 3	21 (17)
CS I + II	77 (62)
CS III	10 (8)
CS IV	37 (30)
**Extranodal involvement**	42 (34)
Lungs	27 (22)
Chest wall	7 (5.6)
Kidney	3 (2.4)
Ovary	3 (2.4)
Bone marrow	2 (1.6)
Central nervous system	1(0,8)
Pleural effusion	42 (34)
Pericardial effusion	36 (29)
Superior vena cava syndrome	52 (42)
Extranodal sites > 1	4 (3)
LDH > ULN	107 (86)
LDH > 3 × ULN	33(27)
IPI score 1	34 (27)
IPI score 2	60 (48)
IPI score 3	26 (22)
IPI score 4	4 (3)
Bulky tumor > 10 mm Max diameter (range) in mm	103 (83)117 (50–300)

*LDH* dehydrogenase lactate, *ECOG* Eastern Cooperative Oncology Group, *IPI* international prognostic index, *ULN* upper limit of normal.

The response assessment was based on WB-CT scans, the additional PET evaluation was performed in 96 (78%) patients: in 68/120 (57%) at the end of chemotherapy and in 66/92 (72%) at the end of radiotherapy.

**Figure 2:**
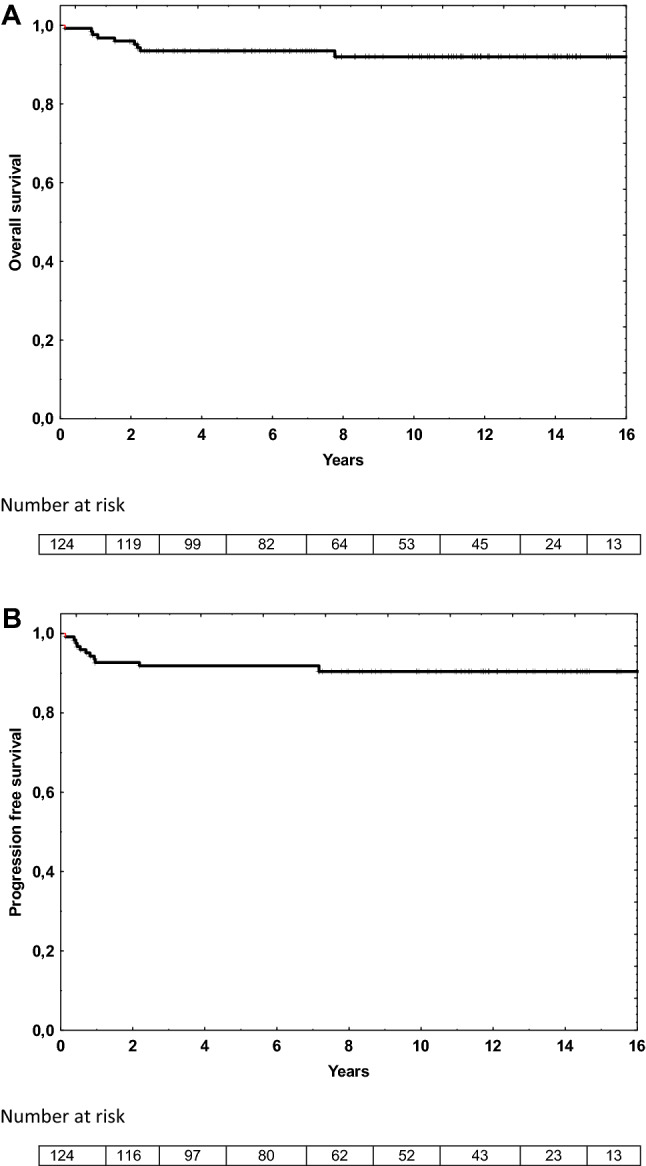
Outcomes based on Kaplan–Meyer analysis: overall survival (**A**) at 5 year was 94% (95% CI 90–98%) and progression free survival (**B**) at 5 year was 92% (95% CI 88–96%). Median follow up 8.5 years.

At the end of chemotherapy ORR in 120 evaluable patients was 97% (78% CR, 19% PR), 4 patients (3%) had progressive disease. With a median (range) follow up of 9 years (1–17), the 5-year OS was 94% (95% confidence interval (CI) 90–98%), (Fig. 2A) and the 5-year PFS was 92% (95% CI 88–96%) (Fig. 2B).

There was no difference in survival for irradiated and non-irradiated patients at CS I–III: the 5-year OS and the 5-year PFS were 100% (95% CI 97–103%) and 99% (95% CI 96–102%) for irradiated patients, respectively, and 99% (95% CI 96–102%) and 96% (95% CI 93–100%) for non-irradiated patients, respectively (p = NS for OS and PFS) (Table 2).

**Table 2:** Overall and progression free survival—univariate analysis.

Characteristics	No (%)	PFS	OS
		p-value	p-value
**Performance status**			
0–2	103 (83)	0.007	0.0012
3–4	21 (17)		
**CS**			
I/II	77 (62)	0.001	0.0010
III/IV	47 (38)		
**CS**			
I–III	87 (70)	0.00009	0.00007
IV	37 (30)		
**LDH**			
Normal	18 (14.5)	0.16	0.20
Elevated	106 (85.6)		
**Pericardial effusion**			
No	88 (71)	0.04	0.05
Yes	36 (29)		
**Pleural effusion**			
No	82 (66)	0.36	0.92
Yes	42 (34)		
**Extranodal site > 1**			
No	120 (97)	0.003	0.0007
Yes	4 (3)		
**IPI**			
0–2	94 (76)	0.001	0.0018
3–5	30 (24)		
**Radiotherapy at CS I–III**			
No	16 (19)	0.23	0.64
Yes	68 (81)		

*CS* clinical stage, *IPI* international prognostic index, *LDH* dehydrogenase lactate, *No* number, *OS* overall survival, *PFS* progression free survival.

Sixty eight patients were evaluated with PET-CT at the end of chemotherapy, 54 (79%) patients achieved mCR and 14 (21%) patients had positive PET-CT: 10 pts had DS 4 and 4 patients had DS 5. The 5-year OS and the 5-year PFS were 96% and 94% in PET-negative patients, respectively, 70% and 70%, in PET-positive, respectively (p = 0.004 for OS and p = 0.01 for PFS). All patients with DS 4 proceeded to consolidative radiotherapy. Out of 4 patients with DS 5, 3 patients started with salvage chemotherapy (R-CHOP or ESHAP) before planned autologous hematopoietic cell transplantation (auto-HCT) procedure and 1 patient had consolidative radiotherapy. At the end of radiotherapy 66 patients were evaluated: 92% of them were in mCR, 4 patients with positive PET reached DS 4, and 1 patient had DS 5. Patients with DS 4 had no progressive disease on follow-up PET scans. The patient with DS 5 underwent high-dose therapy (HDT) following auto-HCT. At the end of radiotherapy the 5-year OS was 100%, regardless of PET results, but the 5-year PFS was 100% vs 80% (p < 0.01) in PET-negative vs in PET-positive patients, respectively.

**Figure 3:**
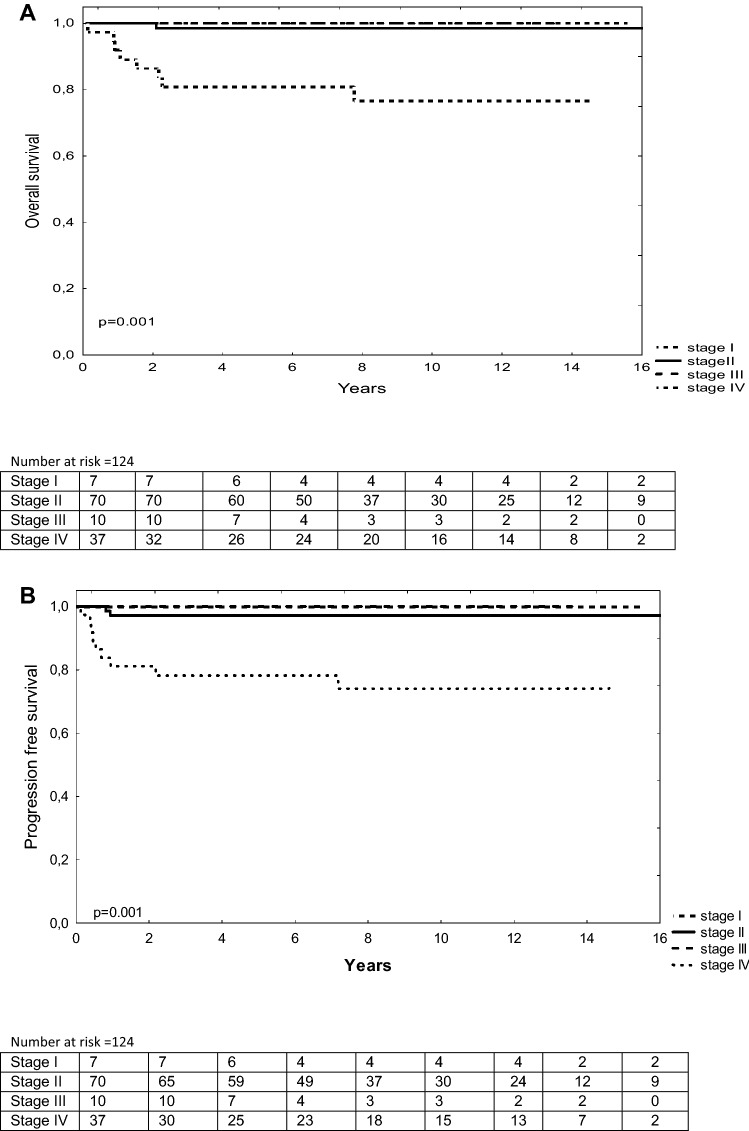
Overall survival (OS) and progression free survival according to clinical stage (CS). OS (**A**) at 5 year was 100% for CS I, 98% for CS II, 100% for CS III and 78% for CS IV. PFS (**B**) at 5 year was 100% for CS I, 98.5% for CS II, 100% for CS III and 81% for CS IV.

**Figure 4:**
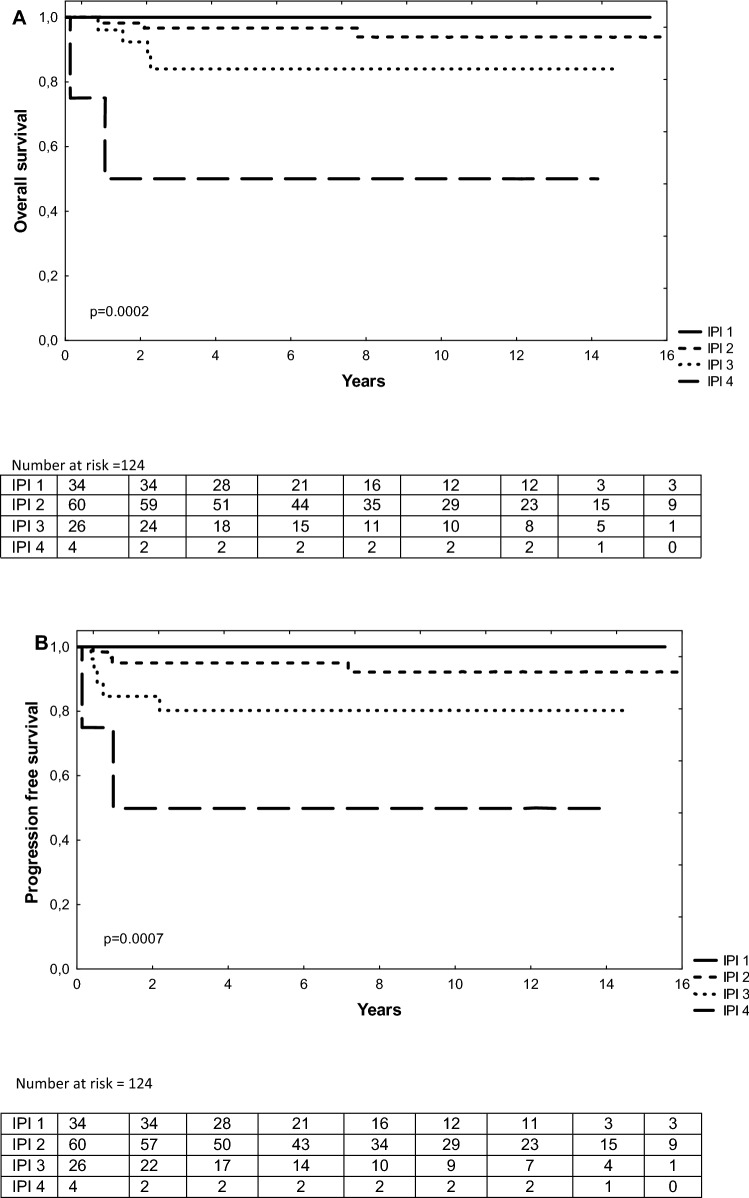
Overall survival (OS) and progression free survival (PFS) according to IPI score. OS (**A**) at 5 year was 100% for IPI 1, 96% for IPI 2, 84% for IPI 3 and 50% for IPI 4. PFS (**B**) at 5 year was 100% for IPI 1, 965% for IPI 2, 80% for IPI 3 and 50% for IPI 4.

Clinical stage IV (Fig. 3), performance status (PS) 3–4, the presence of pericardial effusion, a number of extranodal sites > 1, IPI score 3–4 (Fig. 4) were associated with poorer OS and PFS in univariate analysis (Table 2). The multivariate analysis was not done due to a small number of events.

In total, 8 patients (6%) had treatment failure; 4 (3%) relapsed and 4 had primary refractory disease (3%). All relapses occurred within 1 year after the completion of therapy. None of patients relapsed in CNS. Two patients underwent salvage chemotherapy (R-CHOP-14) and auto-HCT with BEAM conditioning and they are alive. The other 2 patients were not eligible for auto-HCT procedure; 1 received salvage radiotherapy and 1 patient receive palliative chemotherapy (IVAC, CNOP), both died from lymphoma. Out of 4 patients (3%) with refractory disease, 1 patient had unsuccessful consolidative radiotherapy, then he received R-ICE, HDT with auto-HCT, pembrolizumab, and R-CHOP he died due to progressive disease; 1 patient died after second line treatment (CNOP) due to progressive disease; 2 patients underwent auto-HCT with BEAM conditioning, but 1 patient died in CR due to auto-HCT toxicity, second patient received consolidative RT post auto-HCT but he died due to lymphoma. In total, 5 patients with relapsed/refractory disease died from lymphoma, 1 patients because of toxicity related to auto-HCT and 2 patients are alive.

Toxicity of grade 3–4 per treatment cycle and cumulative toxicity for all cycles are listed in Table 3. Pancytopenia was the most often with associated neutropenia grade 3–4 of short duration occurring in all patients. In parallel to neutropenia, infections were more frequent in block A1 with an incidence of 49%, and 20–25% of patients in subsequent cycles. Septic shock and severe pneumonia occurred in 1% and 2% of all cycles, respectively. Thrombocytopenia occurred in more than half of patients with low incidence of bleeding. Mucositis grade 3–4 was the highest in B1 cycle (38% of patients) and decreased to 12–20% of patients in next cycles. Cumulative incidence of neurotoxicity grade 4 (1.3%) included seizures (3 patients) and reversible paresis of the lower extremities probably related to cytarabine infusion (1 patient). Cardiovascular toxicity grade 3/4 included: fatal heart failure after second cycle (1 patient), atrial fibrillation a few days post doxorubicin infusion (1 patient), cardiac arrest during etoposide infusion (1 patient) successfully resuscitated, uncompensated hypertension (1 patient). Other non-hematologic toxicities grade 3/4 occurred in a few patients. 1 patient died because of a heart failure in the course of pericardial effusion.

**Table 3:** Summary of grade 3–4 toxicity of treatment.

	Blocks						All
	A1	B1	C1	A2	B2	C2	
Number of evaluable cycles (%)	124 (100)	122 (100)	121 (100)	118 (100)	116 (100)	114 (100)	715 (100)
Hb < 8.0 g/dL	38 (25)	26 (21)	49 (40)	38 (32)	26 (22)	43 (38)	220 (31)
Neutropenia < 1.0 Giga/L	122 (98)	71 (53)	116 (96)	107 (91)	77 (66)	109 (97)	512 (72)
Platelel < 25 Giga/L	76 (61)	10 (8)	71 (57)	75 (64)	23 (21)	73 (65)	328 (49)
Neutropenic fever	61 (49)	20 (16)	32 (26)	25 (26)	24 (21)	32 (28)	214 (27)
Septic shock	3 (2.4)	0	1 (0.8)	0 (0)	0	3 (2.4)	7 (1)
Pneumonia	5 (4)	2 (1.6)	4 (3.2)	3 (2.4)	0	0	14 (2)
Mucositis grade 3/4	16 (13)	38 (31)	16 (13)	20 (17)	26 (22)	15 (13)	113 (18)
Diarrhea grade 3/4	22 (18)	8 (6)	14 (11)	11 (10)	9 (6.3)	18 (16)	82 (11)
Renal toxicity grade 3/4	1 (0.8)	2 (2)	0 (0)	1 (0.8)	0	0	4 (0.50)
Neurological	1 (0.8)	0	2 (1.6)	2 (1.6)	1 (0.8)	0	6 (0.7)
Cardiac grade 3/4	0	3 (2.4)	1 (0.8)	0	0	0	4 (0.5)
Toxic death	0	1 (0.8)	0	0	0	0	1 (0.8)

Second primary malignancies (SMP) occurred in 3 patients (2.4%) including PMBL follicular lymphoma and hepatic sarcoma diagnosis after 7, 9 and 11 years after therapy, respectively. Late cardiotoxicity (2.4%) involved grade 3 heart failure in 1 patient, and arrhythmia in 2 patients. Seven patients gave birth 7 healthy children.

## Discussion

A prospect of cure for PMBL patients depends on the successful initial therapy because prognosis in case of disease progression or relapse is extremely poor^20–22^. The most common approach is currently R-CHOP^4,5,7–9,23,24^ ± RT or DAEPOCH-R^11,12,23,24^; more intensive protocols^13,15,25,26^ are rarely used. Here we report the long term results of intensive alternating immunochemotherapy protocol GMALL/B-ALL/NHL2002 with high-dose cytarbine and intermediate-dose methotrexate followed by consolidative radiotherapy in 124 PMBL patients. We adopted a Burkitt-type protocol for PMBL patients because of promising preliminary data on activity in PMBL^13^, poor historic outcomes of CHOP chemotherapy at our institution with 5-year OS of 50%^27^, and disappointing results of salvage treatment for relapsed/refractory PMBL patients^20–22^. The advantage of that GMALL/B-ALL/NHL2002 regimen was the attenuated cumulative doxorubicin dose (100 mg/m^2^ for the whole treatment) which could reduce cardiotoxicity in young patients in long term follow up and intermediate dose of methotrexate to decrease risk of CNS relapse. In addition to chemotherapeutic agents normally applied in the first line, GMALL/B-ALL/NHL2002 protocol included ifosfamide, cytarabine and etoposide usually used in salvage treatment. In GMALL/B-ALL/NHL2002 regimen they have been moved to front line therapy to minimize risk of developing resistance to chemotherapy^17^. The GMALL/B-ALL/NHL2002 produced 90% ORR and 80% CR in PMBL patients^28^. After a median follow up of 8.6 years, 73% of patients achieved long-term disease free survival more than 2 years after start of therapy^28^. Our preliminary experience with GMALL/B-ALL/NHL2002 was promising^13,14^. Despite poor risk factors including CSIV, extranodal involvement, or pericardial effusion in 29–34% of patients, the long term remission and survival was achieved in more than 90% of our patients. Relapsed/refractory disease was diagnosed only in 6% of patients. These results are consistent with data from preliminary studies using GMALL/B-ALL/NHL2002 protocol^13,15^ with ORR of 80–100% and long term disease-free survival in 90%^15^. In our patients, relapse or progression occurred within 1 year from the end of treatment. Disease usually recurred at initially involved sites and no CNS relapses were seen. Possibly, the application of intermediate dose of methotrexate and cytarabine, intrathecal prophylaxis contributed to patients’ protection from fatal complications of CNS disease despite most of the patients having at least 3 risk factors for CNS relapse^29^. In other studies involving GMALL/B-ALL/NHL2002 protocol, CNS relapses were not observed either^13,15,28^. In other reports. relapses were reported within 2 years after the end of R-CHOP^6–8^ or DA-EPOCH-R therapy^30^ and CNS relapses occurred in 2–3% of PMBL patients^6–8,30^. Hematologic toxicity of treatment notable for neutropenia and thrombocytopenia was substantial but reversible, and it could be expected with intensive immunochemotherapy. Only one death was considered related to treatment toxicity. Neutropenic fever occurred in 27% of treatment cycles, and there was no death from infection in our patients. Grade 3–4 neutropenia and infections were frequently reported in other studies^15,28^ with TRM due to infections reported in 4.0–8.3% of patients^13,15,28^.

Little is known about late effects of treatment in PMBL with a few secondary primary malignancies recorded after long-term follow up^7,24,31^, as well as occasional late cardiotoxicity^30,32,33^ and infertility^28^. In historical data of published reports secondary malignancies were reported in 1–2% of PMBL patients without established relation to mediastinal radiotherapy^24,25^. Broccoli et al. reported 2 SPM in 98 patients treated with MACOP/B in 20-years follow-up^25^. In our study we recorded only 2.4% of SPM, a rate that could be expected in less intensive therapies.

Cardiotoxicity remains one of significant side effects of standard R-CHOP therapy. The Danish Lymphoma registry examined cardiotoxicity in patients treated with R-CHOP/R-CHOEP. Authors showed that the risk of cardiotoxicity increased depending of number of cycles and the cumulative 5 year risk of congestive heart failure (CHF) after 3–5 cycles was 4.6% and > 6 cycles 7.9%^32^. Another study on cardiotoxicity in young high risk DLBCL patients treated with R-CHOEP14 demonstrated that the cumulative 5-year risk of CHF with all causes mortality as the competing risk was 17%^33^. Data focused on cardiotoxicity in PMBL patients treated DA-EPOCH-R are discrepant. Dunleavy et al. did not observe any cardiac toxic effects^11^, Giulino-Roth et al. showed that the cardiac abnormalities occurred in 15.6% of paediatric patients and in 13.1% of adults after DA-EPOCH-R in long term follow up, additionally 28% of all had thrombolytic complications^30^. In our cohort only 3% of GMALL treated patients experienced acute cardiotoxicity; the late cardiac toxicity occurred in an even lower number of patients (2.4%). This could be probably likely related to limited dose of doxorubicin(100 mg/m^2^). Despite high intensity of GMALL protocol and application of consolidative RT to 72% of patients, both SPMs and incidence of cardiotoxic events remain low at a median of 9 year follow up.

The role of consolidative radiotherapy after induction treatment is controversial. Radiotherapy is believed to improve response to systemic treatment in PMBL from PR to CR and may results in long-term survival in 92–94% of patients^10,25,34^. A retrospective evaluation of 250 PMBL patients treated between 2001 and 2011 demonstrated advantage of combined chemotherapy and radiotherapy over chemotherapy alone (5-year OS of 90% vs 79%, p = 0.02)^34^. The prospective randomized UNFOLDER study of 131 PMBL patients treated with R-CHOP14 vs R-CHOP21 ± irradiated (82 pts) showed no differences in results between these two regimens and between irradiated and no irradiated arms (PFS 95% vs 90%, p = 0.253, OS 98% vs 96%, p = 0.636). Only patients who achieved PR after chemotherapy had a significant benefit from consolidative radiotherapy^10^. Recently, PET-guided approach has been increasingly adopted with the use of RT limited to EOT-PET-positive patients^35^. Several studies support this strategy that PET negative patients may be safely observed^11,36–39^. The IELSG-37 phase III study is expected to establish whether RT is of any benefit in this indication^11,35,39^. In our study, most of the patients were irradiated, but there was no apparent survival benefit of RT in patients with CSI-III who received RT over those who were not irradiated according to the decision of the treating physician. Given the extended time span when patients were treated, including early period when PET scan was not routinely available, referral to RT was not consistently guided by PET in our patients.

Several studies indicate 10–20% of refractory disease^4,9,20,21,23^, associated with dismal prognosis. A few studies support use of anti-PD1 antibodies in this conditions ± brentuximab vendotin^40,41^ and CAR-T therapy^42^ is particularly promising, however access to these modern therapies is limited. On the other hand there are no reliable markers predictive for the poor outcomes^,^^4,7,9,20–22^. The IPI risk classification is of a little help due to young age and early stage of disease in most patients. Nevertheless, the high IPI, pleural/pericardial effusion, combination CSIV with bulky disease, and high LDH level are predictive for worse outcomes in less intensive therapies^9,43^. In our study, we found some unfavourable factors for survival in univariate analysis but we could not confirm their predictive value in multivariate analysis because of a small number of events.

The limitations of our study include a single-center experience and some changes in practice over an extended period of time when patients were treated. For instance, access to PET-CT examination was limited in the initial years of study, and the method of response evaluation was not uniform as were criteria for referring patients for consolidative radiotherapy. For that reason no conclusions on the role radiotherapy can be based on our data. The value of this study is a substantial patient population and an uniform treatment protocol involving all patients.

In conclusion, results of this study indicate high efficacy and very low rate of treatment failure in a substantial group of patients with PMBL. Of note is the absence of secondary CNS involvement in our group of patients. The advantage of the protocol is a relatively low dose of doxorubicin which may contribute to reduced cardiotoxicity and interrmediate dose of methotrexate potentially protecting patients from CNS relapse. Acute hematologic toxicity was substantial but manageable and reversible. Given the prolonged follow up in this study there is no signal of remarkable late effects of treatment. Consolidative radiotherapy does not seem to have an impact on survival in patient who achieve complete remission, but our data is too limited to confirm this observation. GMALL/B-ALL/NHL2002 intensive immunochemotherapy can be a vital option for patients with high risk and advance stage or with high risk of cardiotoxicity.

## Data Availability

All dataset and material used and analyzed during current study are available from corresponding authors on reasonable request.
